# Ultrasound-Guided versus Fluoroscopy-Guided Deep Cervical Plexus Block for the Treatment of Cervicogenic Headache

**DOI:** 10.1155/2017/4654803

**Published:** 2017-02-23

**Authors:** Qing Wan, Haiyun Yang, Xiao Li, Caina Lin, Songjian Ke, Shaoling Wu, Chao Ma

**Affiliations:** ^1^Pain Treatment Centre of Department of Rehabilitation Medicine, Sun Yat-sen Memorial Hospital, Sun Yat-sen University, Guangzhou, Guangdong Province, China; ^2^Department of Medical Ultrasonics, Sun Yat-sen Memorial Hospital, Sun Yat-sen University, Guangzhou, Guangdong Province, China

## Abstract

*Objective*. The aim of this study was to compare the efficacy of ultrasound-guided deep cervical plexus block with fluoroscopy-guided deep cervical plexus block for patients with cervicogenic headache (CeH).* Methods*. A total of 56 patients with CeH were recruited and randomly assigned to either the ultrasound-guided (US) or the fluoroscopy-guided (FL) injection group. A mixture of 2–4 mL 1% lidocaine and 7 mg betamethasone was injected along C_2_ and/or C_3_ transverse process. The measurement of pain was evaluated by patients' ratings of a 10-point numerical pain scale (NPS) before and 2 wks, 12 wks, and 24 wks after treatments.* Results*. The blocking procedures were well tolerated. The pain intensity, as measured by NPS, significantly decreased at 2 wks after injection treatment in both US and FL groups, respectively, compared with that of baseline (*P* < 0.05). The blocking procedures had continued, and comparable pain relieving effects appeared at 12 wks and 24 wks after treatment in both US and FL groups. There were no significant differences observed in the NPS before and 2 wks, 12 wks, and 24 wks after treatment between US and FL groups.* Conclusions*. The US-guided approach showed similar satisfactory effect as the FL-guided block. Ultrasonography can be an alternative method for its convenience and efficacy in deep cervical plexus block for CeH patients without radiation exposure.

## 1. Introduction

Cervicogenic headache (CeH) is a common diagnosis for patients with unilateral referred pain to the head from the upper cervical spine [1]. The prevalence of CeH has been estimated at up to 20% among patients with chronic headaches [2]. Treatment strategies for CeH are wide and varied, such as medication, physical therapy, acupuncture, manipulation, transcutaneous electrical nerve stimulation, pulsed radiorefrequency, injections, and surgery [3, 4]. The majority of patients preferred to choose the noninvasive strategies, including physical and manual therapies, activity modification, and various medication trials before considering anesthetic blocks [1, 2, 5–7]. There is an ongoing need for evidences of long-term effect and high-quality randomized controlled trail investigations for these strategies.

The C_2_-C_3_ zygapophysial and atlantoaxial joints are believed to be primary causes for CeH and the C_2_ nerve may be more susceptible to injury by aseptic inflammation or compression than the other structures [3]. Thus, several local anesthetic techniques have been used in treating CeH, including occipital nerve blocks, cervical facet joint blocks, and cervical epidural injections [8–11]. Real time fluoroscopy imaging guidance is frequently required to ensure correct needle placement [12–14]. However, repeated exposure to radiation is a great threat to the health of physicians. Ultrasound-guided injections have been described recently in the literatures, and the absence of radiation exposure, equipment affordability, and bedside setting are advantages of ultrasonography compared with traditional radiological imaging [15–18].

The objective of this study was to evaluate the efficacy of ultrasound-guided deep cervical plexus block for patients with CeH in comparison with fluoroscopy-guided block through a prospective, randomized, single-blind clinical trial.

## 2. Materials and Methods

There were 84 eligible patients with CeH and 60 patients participated in this randomized, single-blind study. They were enrolled and then randomly assigned into two groups according to a random number table and underwent ultrasound-guided (US, *n* = 30) or fluoroscopy-guided (FL, *n* = 30) C_2_ and/or C_3_ transverse process injection, respectively, in the pain center of Sun Yat-sen Memorial Hospital from July 2011 to January 2014. The study was approved by our university and all patients gave informed consent before this study.

The patients had experienced screening that included medical history and physical examination followed by X-ray, CT scan, or MRI of the cervical spine. All patients were diagnosed with CeH according to the diagnostic criteria of Sjaastad et al. [19]: (1) unilateral or bilateral pain starting in the neck and radiating to the frontotemporal region, (2) pain aggravated by neck movement, (3) restricted cervical range of motion, (4) joint tenderness in at least one of the joints of the upper cervical spine (C_2_-C_3_), and (5) headache frequency of at least 1 per week over a period greater than 3 months. In this study, we chose the patients with unilateral pain. Our exclusion criteria included (1) pathologic fracture, cancer, and other diseases of the cervical vertebra, (2) headache caused by nervous system diseases, or other factors.

### 2.1. US-Guided Blockade Procedures

**Figure 1:**
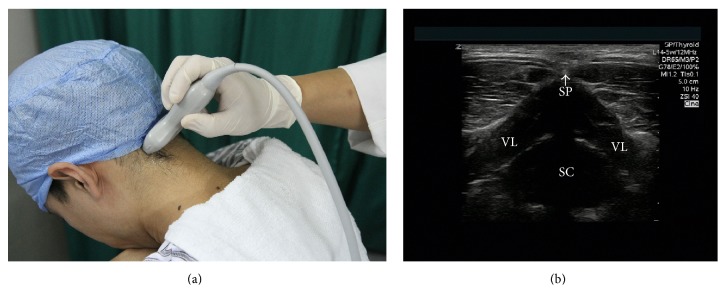
(a) The position of the patient. (b) Transverse ultrasound view of C_2_ spinal process (SP), spinal canal (SC), and vertebral lamina (VL).

**Figure 2:**
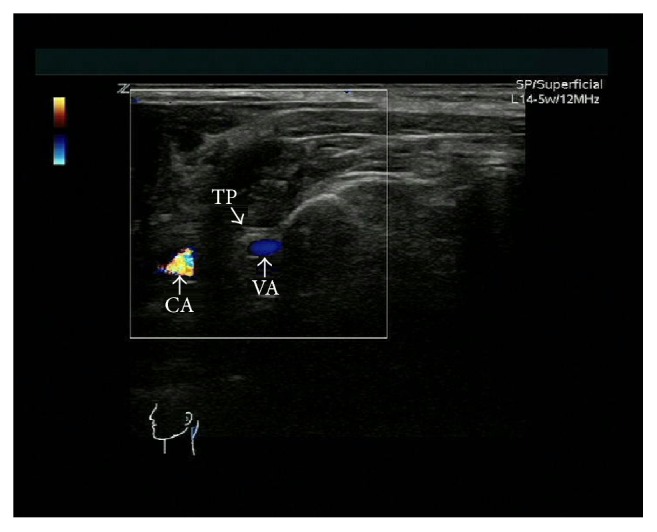
Transverse ultrasound view of C_2_ transverse process (TP), vertebral artery (VA), and carotid artery (CA) (left side).

**Figure 3:**
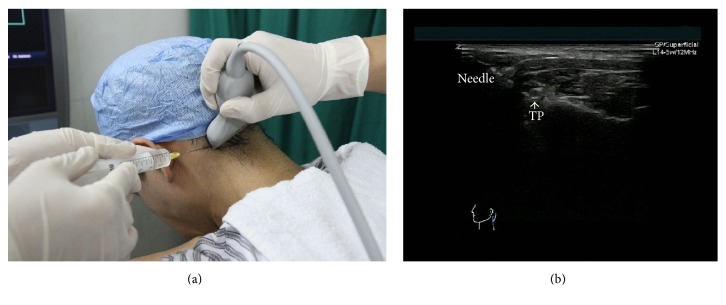
(a) Ultrasound-guided C_2_ transverse process block (left side). (b) The needle is targeted just lateral to the transverse process (TP) (left side).

The patients were placed in sitting position with the neck bending forward and the head was supported (Figure 1(a)). The selection of the site for injection was determined by first having the patient identify the more painful side [8, 11]. An Esaote MyLab60 (Esaote, Italy) ultrasound machine equipped with a multifrequency linear probe (4–13 MHz) was used. The skin was prepared with betadine and draped in the usual sterile fashion at the region of injection. The ultrasound probe was also sterilized with betadine. On the transverse section, the spinal process of C_2_ or C_3_ was identified on the ultrasound image (Figure 1(b)). Then, the probe was moved laterally from midline and the posterior elements, including layers of muscle, lamina, interlaminar space, facet joint, C_2_ or C_3_ transverse process, and vertebral artery that were identified on the transverse sonograms at the same level (Figure 2). A 22-gauge 3.5-inch spinal needle was inserted to C_2_ or C_3_ transverse process (Figures 3(a) and 3(b)). When the needle reached the bone, after negative aspiration, total 2–4 mL of 1% lidocaine with 7 mg of betamethasone was injected in slow speed while maintaining communication and questioning of the patient to ensure that the needle was not intravascular or intrathecal. If the headache was unilateral in nature, the block was performed only on the affected side (C_2_ and/or C_3_). If necessary, the patients would receive the second injection in one-week interval after the first injection. All procedures were performed in the same fashion by C. M and HY. Y.

### 2.2. FL-Guided Blockade Procedures

**Figure 4:**
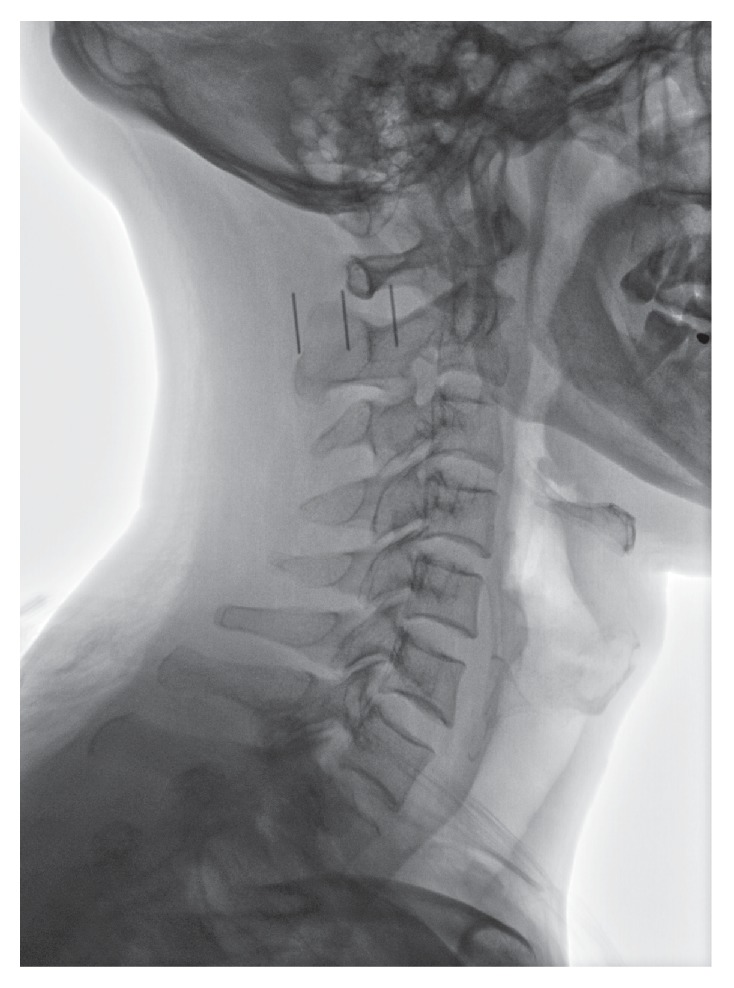
The C_2_ or C_3_ spinal process is identified on the lateral view under fluoroscopy guidance with external landmarks.

**Figure 5:**
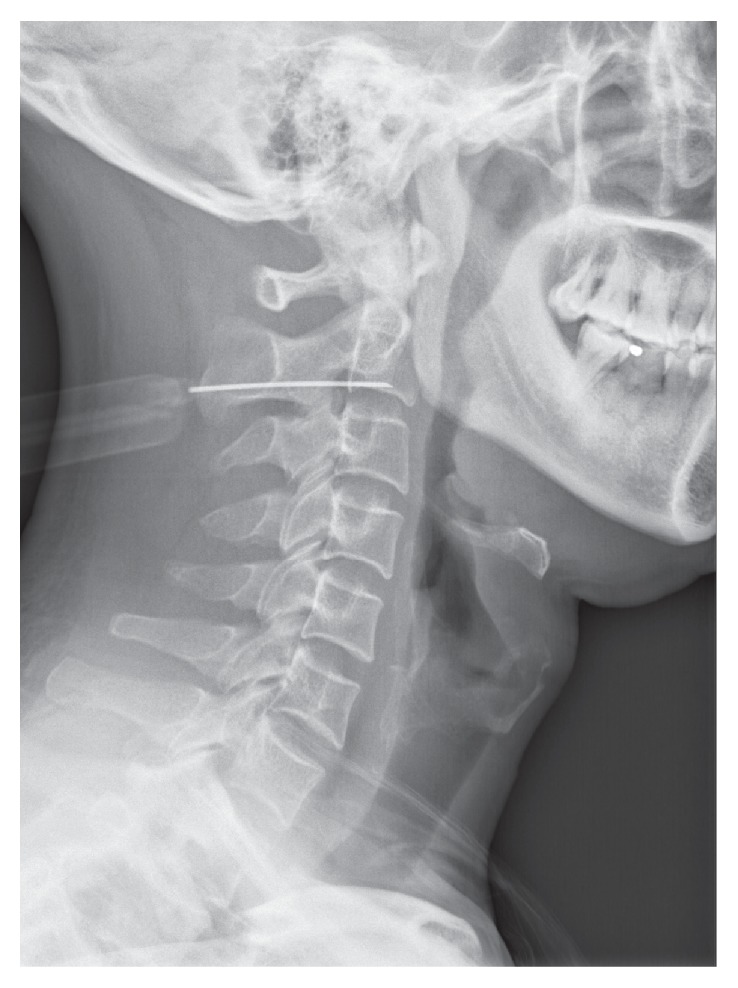
The position of needle is confirmed in the lateral projection.

The patients were placed in supine and, using external landmarks, the C_2_ or C_3_ spinal process was identified on the lateral view (Figure 4). After routine skin preparation and sterilization, a 22-gauge 3.5-inch spinal needle was inserted into the lateral recess of C_2_-C_3_ and its position was confirmed in the AP and lateral projections (Figure 5). All procedures were performed by the same physician Q. W. In one week at the return visit, the physician applied the second block treatment if necessary, depending on the symptom of patients.

### 2.3. Outcome Assessment

The measurement of pain was evaluated by patients' ratings of a 10-point numerical pain scale (NPS, from 0, no pain, to 10, the worst pain). Pain was assessed before the injection and the return visit was conducted in 2 weeks after treatment to exclude any major complications. All the patients were followed up at 12-week and 24-week intervals with telephone consultation and visits to the pain clinic.

### 2.4. Data Analysis

The pain intensities were measured on a 0–10 NPS. The characteristics of the US and FL groups such as sex, age, and duration of pain were compared by the *χ*^2^ test and Mann–Whitney *U* method. At each time point of injection, the NPS was compared by repeated-measures analysis of variance, and the Bonferroni correction was conducted for post hoc comparison. Statistical analysis was carried out using SPSS 16.0 (SPSS Inc., Chicago, IL, USA). Values are expressed as means ± SD and a* P* value of less than 0.05 was considered to be statistically significant.

## 3. Results

### 3.1. Patient Characteristics

**Figure 6:**
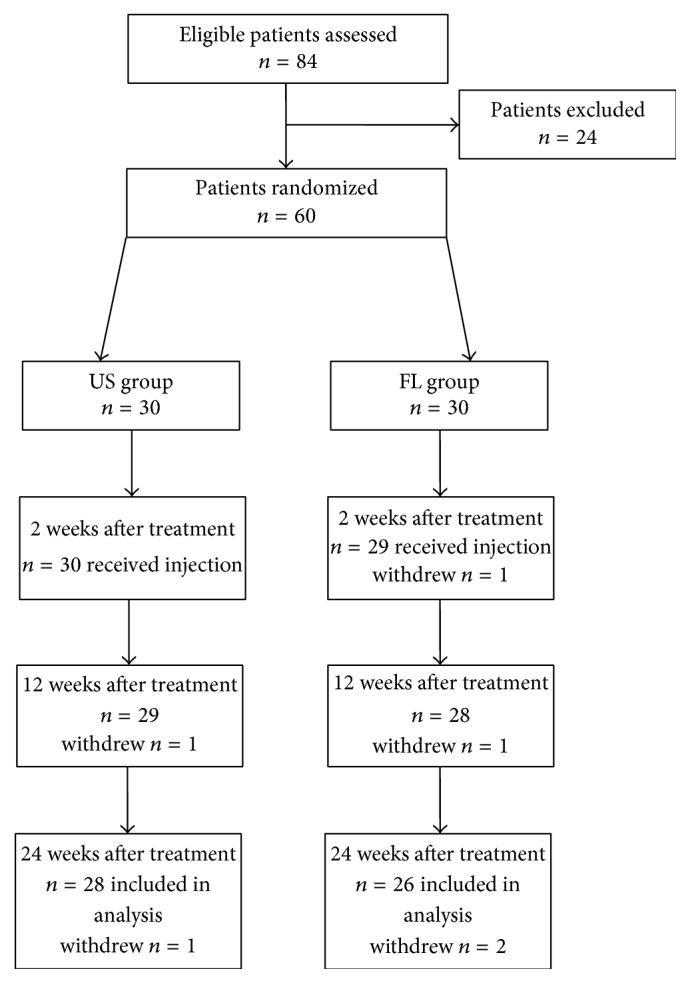
Schematic presentation of participant flow.

A total of 84 consecutive patients were assessed for eligibility during this study, with 60 of them being enrolled and 54 patients completing the study. A flow diagram with patient selection and follow-up is presented in Figure 6. In US group, two patients lost track in the follow-up and the other patients (*n* = 28, 7 males and 21 females) completed the injection and 12 patients received the second injection treatment. In FL group, one patient chose other noninvasive interventions and three patients were lost in the follow-up because of refusal to radiation exposure; a total of 26 patients (5 males and 21 females) completed this study and one-half of them received the second block. The patients had comparable pain duration for 10.06 ± 3.47 months as that of US group. The majority of location of the block was the C2 transverse process (71.43% in US group and 80.77% in FL group). The demographic data of patients are present in Table 1.

### 3.2. Treatment Effects between the Approaches

The blocking procedures were tolerable for all the patients during the treatment. However, 3 patients in US group felt dizzy immediately after injection; the symptom disappeared when they lay down on the bed for several minutes. A total of 4 patients (2 in US group and 2 in FL group) complained of neck and shoulder uncomfortableness. None of the patients in this study had any treatment-related severe complications. There were 25 patients (12 in US group and 13 in FL group) who received the second injection according to their symptoms at the return visit.

There were no significant differences observed in the NPS before and 2 wks, 12 wks, and 24 wks after the injections between US and FL groups (Table 2). The pain intensity, as measured by NPS, significantly decreased at 2 wks after injection treatment in both US and FL groups, respectively, compared with that of baseline (*P* < 0.05). The blocking procedures had continued, and comparable pain relieving effects appeared at 12 wks and 24 wks after treatment in both US and FL groups (Table 2), the NPS score significantly decreased 12 wks and 24 wks after treatment compared with that at baseline (*P* < 0.05) and at 2 wks after injection (*P* < 0.05). It indicated that steroid injection guided by either ultrasound or fluoroscopy had sustained pain-relieving effect for CeH patients.

## 4. Discussion

Cervicogenic headache is a common clinical syndrome and few conservative managements lead to satisfactory clinical efficacy. There are multiple causes of CeH, including the C_2_-C_3_ and C_3_-C_4_ z-joints, A-A joint, C_2_-C_3_ intervertebral disk, A-O joint, and the greater or lesser occipital nerve [1]. Anatomically, the main dorsal ramus of C_2_ becomes the greater occipital nerve after passing the posterior aspect of the C_1/2_ facet joint. The C_2_ and C_3_ ventral rami become the lesser occipital nerve, and the C_3_ dorsal ramus becomes the third occipital nerve [1, 2, 8, 11]. The pain of CeH most commonly originates from the C_2/3_ facet joints [20]. In this study, US or FL-guided C_2_ and/or C_3_ transverse process steroid injections were applied to 54 patients with CeH and comparable pain relief effect was observed. It has been frequently demonstrated that blockade of the greater occipital nerve, the lesser occipital nerve, the stellate ganglion, and other various blocking treatments are effective strategies for CeH. Zhou et al. reported that, of the 28 CeH patients, 26 of them had abnormalities in the cervical spine below the C_1/2_ and C_2/3_ levels and C_1/2_, C_2/3_ facet joint injections and C_2_, C_3_ spinal rami blocks were effective and well tolerated by the patients [8]. Anthony reported that injection of methylprednisolone into the greater and lesser occipital nerve region could relieve headache completely in 169 out of 180 patients for a duration of 10–77 days [21]. Our results also showed sustained pain relief effect of steroid injections for as long as 6 months; however, these results were inconsistent with those of Goldberg et al. [11]. The authors applied FL-guided deep cervical plexus block to 39 patients with CeH; the mean pain scores were significantly lower than baseline at 3 months but by 6 months the pain had returned to pretreatment levels. One possible explanation could be the difference in inclusion criteria; the study of Goldberg et al. recruited patients suffering from atypical headaches for longer duration of 4-5 years, while it were typical CeH patients with the symptoms only for 5.7–13.5 months in our study. Another factor contributing to the varied results could be frequency of injection treatment for patients with longer and shorter history. Our patients with a history for 5.7–13.5 months received 1-2 blockade treatments, while the great majority of patients (87%) with a history for more than 4 years in their study only had a median treatment frequency for 2 injections. For patients with longer history of CeH, more treatment sessions might be considered for longer pain relief effect.

The C_2_ and/or C_3_ transverse process block technique is a modification of the deep cervical plexus block that has been utilized to provide anesthesia to the head and neck region [15]. This technique is relatively safe since the cervical foramen is not entered and vertebral artery (Figure 2) can easily be detected by ultrasonography. Although this block occurred more peripherally compared to z-joint and transforaminal block, our results demonstrated that the blocking strategy has efficient pain relief effect. The analgetic solution spreads within the cervical region to adjacent levels since the paravertebral space communicates freely [22], which allows local injection at one level that can achieve the same beneficial effect.

The FL-guided injection procedure is most commonly used in treating pain-associated diseases to ensure correct needle placement. However, radiation exposure was unavoidable, and there is a risk of vertebral artery injury. The US-guided method has several advantages in comparison with the FL-guided method. Gofeld et al. explored US-guided lumbar transforaminal and z-joint injection techniques for patients with spinal pain, and their studies demonstrated that US-guided injections are accurate and feasible [23, 24]. It has also been reported that US-guided selective nerve root block and caudal injection for low back pain are feasible, easy-to-perform, and effective procedures [15, 18]. Ultrasonography may be a viable alternative to fluoroscopy or computed tomography as a guidance method, because it overcomes the disadvantages of radiation exposure and poor vascular display. Furthermore, the device of ultrasonography is portable and convenient to be used at bedside for patients with severe pain.

The main limitations of the study are as follows: first, the sample size was small; thus, clinical findings still need to be confirmed with larger clinical trials. Second, this study was not a double-blind control study. It was difficult to conduct a double-blind control study with nontraditional modalities such as ultrasound and fluoroscopy. Additional investigations may be needed to improve the limitations.

In summary, deep cervical plexus block in C_2_ or C_3_ transverse process for treating patients with cervicogenic headache provided significant and prolonged pain relief (6 months' follow-up). The US-guided approach showed similar satisfactory effect as the FL-guided block. Ultrasonography can be an alternative method for its convenience and efficacy in deep cervical plexus block without radiation exposure.

## Figures and Tables

**Table 1:** General characteristics of the patients.

	US (*n* = 28)	FL (*n* = 26)	* P*
Age	49.2 ± 10.3	47.6 ± 9.7	0.419
Gender			0.747
Male	7	5	
Female	21	21	
Duration (mos)	9.35 ± 3.68	10.06 ± 3.47	0.725
Pain side			0.781
Left	10	11	
Right	18	15	
Number of injections			0.785
1	16	13	
2	12	13	
Injection position			0.530
C_2_	20	21	
C_3_	8	5	

**Table 2:** Comparison of the NRS before and after treatment.

	US	FL
Baseline	7.61 ± 1.12	7.50 ± 1.06
2 wks	3.45 ± 0.54^a^	3.40 ± 0.58^a^
12 wks	2.41 ± 0.62^a,b^	2.39 ± 0.68^a,b^
24 wks	2.36 ± 0.56^a,b^	2.43 ± 0.59^a,b^

^a^Significantly different from the baseline at *P* < 0.05.

^b^Significantly different from 2 wks after treatment at *P* < 0.05.
